# ^18^F-FDG PET/CT-based gross tumor volume definition for radiotherapy in head and neck Cancer: a correlation study between suitable uptake value threshold and tumor parameters

**DOI:** 10.1186/1748-717X-5-76

**Published:** 2010-09-02

**Authors:** Chia-Hung Kao, Te-Chun Hsieh, Chun-Yen Yu, Kuo-Yang Yen, Shih-Neng Yang, Yao-Ching Wang, Ji-An Liang, Chun-Ru Chien, Shang-Wen Chen

**Affiliations:** 1 Department of Nuclear Medicine and PET Center, China Medical University Hospital, Taichung, Taiwan; 2 Department of Radiation Oncology, China Medical University Hospital, Taichung Taiwan; 3 College of Medicine School, China Medical University, Taichung, Taiwan; 4 College of Medicine School, Taipei Medical University, Taipei, Taiwan; 5 Department of Biomedical Imaging and Radiological Science, China Medical University, Taichung, Taiwan

## Abstract

**Background:**

To define a suitable threshold setting for gross tumor volume (GTV) when using ^18^Fluoro-deoxyglucose positron emission tomography and computed tomogram (PET/CT) for radiotherapy planning in head and neck cancer (HNC).

**Methods:**

Fifteen HNC patients prospectively received PET/CT simulation for their radiation treatment planning. Biological target volume (BTV) was derived from PET/CT-based GTV of the primary tumor. The BTVs were defined as the isodensity volumes when adjusting different percentage of the maximal standardized uptake value (SUVmax), excluding any artifact from surrounding normal tissues. CT-based primary GTV (C-pGTV) that had been previously defined by radiation oncologists was compared with the BTV. Suitable threshold level (sTL) could be determined when BTV value and its morphology using a certain threshold level was observed to be the best fitness of the C-pGTV. Suitable standardized uptake value (sSUV) was calculated as the sTL multiplied by the SUVmax.

**Results:**

Our result demonstrated no single sTL or sSUV method could achieve an optimized volumetric match with the C-pGTV. The sTL was 13% to 27% (mean, 19%), whereas the sSUV was 1.64 to 3.98 (mean, 2.46). The sTL was inversely correlated with the SUVmax [sTL = -0.1004 Ln (SUVmax) + 0.4464; R^2 ^= 0.81]. The sSUV showed a linear correlation with the SUVmax (sSUV = 0.0842 SUVmax + 1.248; R^2 ^= 0.89). The sTL was not associated with the value of C-pGTVs.

**Conclusion:**

In PET/CT-based BTV for HNC, a suitable threshold or SUV level can be established by correlating with SUVmax rather than using a fixed threshold.

## Introduction

^18^Fluoro-deoxyglucose positron emission tomography (^18^F-FDG PET) has been shown to improve the staging of head and neck cancer (HNC) [1-5]. ^18^F-FDG PET after definitive radiotherapy (RT) has also been shown to have a good negative predictive value in patients with HNC [6,7]. The use of ^18^F-FDG PET in RT represents an expansion of this already interdisciplinary process to include information on the biologic status of tumors, which is complementary to conventional computed tomogram (CT) images and may result in target volumes that contain proliferating tumor burden. Several institutions have investigated the value of ^18^F-FDG PET in tumor target delineation for HNC [8-12]. While CT remains the gold standard for delineation of tumor volumes for RT planning, these studies reported PET overlay on CT has shown to have some impact the gross target volume (GTV), decrease inter-observer variability and change the treatment planning. However, when a radiation oncologist contours the GTVs on fused PET and CT images at the radiation treatment planning (RTP) workstation, a problem is emerged in setting the threshold for the PET images. The volume of the GTVs on the PET images can be easily altered by simply adjusting the threshold setting. Despites several investigations declared PET-based target delineation results in a change in the gross tumor volume (GTV) compared to CT-based GTV [13-17], some standards should be followed for ^18^F-FDG-based delineation of tumor boundaries when comparing PET-based target volume with conventional CT-based tumor volume [18]. One study used phantoms of a known size in an attempt to define a standard threshold cutoff in ^18^F-FDG PET voxel values [19]. This study suggested that the threshold can be set at 42% of the maximum uptake, though the study considered only lesions in the size range of 0.4 to 5.5 mL, a range in which threshold levels are extremely sensitive.

The published methods based on a threshold determined as a percentage of the maximal standardized uptake value (SUVmax) have used values ranging from 15% to 50% for lung cancer [13-17,20-23]. In HNC series, there was a great variation of validated standardized methods for setting this threshold [5,8-12]; these include using the absolute standardized uptake value (SUV) (i.e., GTV = SUV of > 2.5), using percentages of the SUVmax (i.e., GTV = volume encompassed by > 50% the SUVmax), or ignoring the threshold setting and simply contouring the CT volume corresponding to the visually identified lesion. Three studies have investigated the optimal threshold by different method in target delineation [24-26], but their results were not consistent. To reduce intra-observer or inter-observer variability in GTV delineation using PET, there is a need to conduct another study to clarify this issue.

We hypothesized that a suitable threshold level of ^18^F-FDG PET can be obtained by certain tumor-related parameters when defining GTV for HNC. Thus, this study was conducted to evaluate the appropriateness of the percentage threshold method or other approaches by using PET/CT simulation in determining the suitable threshold level for the best volumetric match for GTV. The PET data of the PET/CT image was only used for CT-based GTV comparison but not for seeking metastatic disease or for changing the radiation treatment strategy.

## Methods

### Patient population

After approval by local institutional review board (number: DMR98-IRB-067), a cohort of 15 fresh HNC patients with a histological proof of squamous cell carcinoma, who would undergo definitive concurrent chemoradiotherapy with an intensity-modulated radiotherapy technique (IMRT) at China Medical University Hospital, were enrolled in this prospective study. The median age was 46 years (range, 36-70 years). Thirteen patients were men and two were women. They received a pretreatment PET/CT for RT planning. No patient was known to have a history of diabetes and all had a normal serum glucose level before taking the PET/CT image. The characteristics of the 15 patients are listed in Table 1.

**Table 1:** Patient's characteristics and their volumetric and PET/CT data

Patient	Tumor type (AJCC stage)	C-pGTV (mL)	SUVmax	BTV (mL) 10% TL	BTV (mL) 20% TL	BTV (mL) 30% TL	BTV (mL) 40% TL	BTV (mL) 50% TL	sTL	sSUV
1	HPC (T2N2)	43.3	30.6	47.9	35.5	30	25.4	19.2	13%	3.98
2	OPC (T4N1)	75.4	17.2	92.1	42.8	31.6	24.8	18.2	16%	2.75
3	NPC (T4N2)	110.2	17	139.2	77.3	59.3	47	37.5	15%	2.55
4	NPC (T3N1)	30.4	14.6	40.7	22.7	14.6	8.3	2.8	15%	2.19
5	NPC (T1N1)	14.8	15.7	36.6	12	7.2	5.4	4.2	17%	2.21
6	OPC (T3N1)	47.7	24.1	75.8	33.3	21.1	14.1	9.9	14%	3.37
7	NPC (T4N2)	38.7	8.0	-	60	30.8	17.4	12.3	27%	2.16
8	NPC (T3N2)	44.6	17.0	64.7	33.6	24.5	17.5	13.6	15%	2.55
9	NPC (T2N1)	12.8	7.8	39.6	13	5.6	4	2.4	21%	1.64
10	NPC (T4N1)	35.5	8.2	80.7	44	21.9	10	5.6	23%	1.89
11	NPC (T1N1)	9.6	9.0	37.6	13.8	4.9	2.2	1.2	23%	2.07
12	NPC (T3N3)	27.8	17.5	49.9	13.7	9.2	5.3	3.2	14%	2.45
13	NPC (T3N2)	37.1	12.9	67.4	37.9	20.6	12	9.5	20%	2.57
14	NPC (T2N2)	22.4	8.1	-	35.6	14.8	9.2	5.9	27%	2.19
15	HPC (T2N1)	14.3	12.2	46.8	14.5	5.2	3.1	2.3	20%	2.44

Abbreviation: NPC: nasopharyngeal cancer; OPC: oropharyngeal cancer; HPC: hypopharyngeal cancer; C-pGTV: CT-base primary gross tumor volume; BTV: biological target volume from PET/CT-base primary gross tumor volume; TL: threshold level; sTL: suitable threshold; sSUV: suitable SUV.

### PET-CT image acquisition

All patients were asked to fast for at least 4 hours before ^18^F-FDG PET/CT imaging. Approximately 60 minutes after the administration of 370 MBq of ^18^F-FDG, simulation images were taken by PET/CT scanner (PET/CT-16 slice, Discovery STE, GE Medical System, Milwaukee, Wisconsin USA). During the uptake period, patients seated in a comfortable chair and were asked to rest. Whole body PET/CT images were taken first. The procedure did not required immobilization device and take approximately 30 minutes to position the patient and to acquire both the CT and PET data in total. CT images were obtained at 120 kVp and variable mA (AutomA technique) with 3.75-mm slice. The PET data were reconstructed by application of the CT-based attenuation correction and iterative reconstruction algorithm. Immediately after whole body PET/CT images, patients were simulated in a RT set-up position on the PET/CT scanner table with a head and neck immobilization device. An allocated PET/CT imaging field was taken from the base of the skull to upper thorax. The images were electronically transferred from the PET/CT workstation via DICOM3 to the RTP (Eclipse version 8.1, Varian Medical System Inc, CA, USA) in the department of radiation oncology. The workstation provided the quantification of FDG uptake in terms of SUV. Nuclear medicine physicians identified the locations and values of SUVmax for all the primary tumors. This procedure is routinely used on the PET/CT workstation for diagnostic readings, and it allows for definition of threshold level and reproducible contouring of hypermetabolic areas.

### Delineation of CT-based tumor volume

On the basis of axial CT images, contouring of the tumor volume and normal and critical structures was performed without knowledge of the PET results in an effort to reduce bias. Radiation oncologists then delineated the primary gross tumor volume (pGTV) and the metastatic lymph node volume (nGTV). Neck lymph nodes were considered pathological when their smallest axis diameter was > 1 cm. The volumes of all tumors were measured by outlining the lesion on each image if it was visible. No attempts were made to differentiate the tumors from any related edema. The tumor volumes were contoured and the volumes calculated using the same planning system. To reduce inter-observer variations, at least 2 different radiation oncologists carried out the contouring of the tumors for each patient. When the calculated values for any volume varied by more than 10%, an average of the readings was used as the measured volume. When the variation exceeded 10%, contouring and measurement were repeated by 3^rd ^radiation oncologist to correct any bias. In brief, the CT-based primary gross tumor volume would be finally confirmed by at least two radiation oncologists, and abbreviated as C-pGTV. This procedure was addressed in our previous report [27].

### Volumetric match between PET-CT-based GTV and CT-based GTV

After the completion of the C-pGTV contouring in RTP system, the radiation oncologists reviewed the consistency of PET/CT images with nuclear medicine physicians. They also reconfirmed the allocated point of the SUVmax within the tumors.

**Figure 1:**
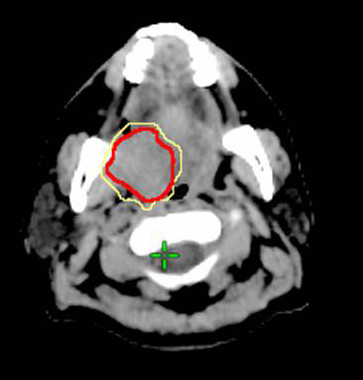
**The biological target volume (BTV) of the primary tumor was determined when using 10% isodensity volumes (yellow line)**. CT-based GTV was outlined by red line.

**Figure 2:**
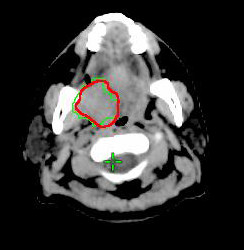
**The BTV of the primary tumor was determined when using 15% isodensity volumes (green line)**. CT-based GTV was outlined by red line.

**Figure 3:**
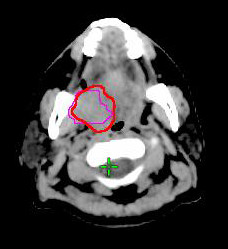
**The BTV of the primary tumor was determined when using 20% isodensity volumes (pink line)**. CT-based GTV was outlined by red line.

Biological target volume (BTV) was derived from PET/CT-based GTV of the primary tumor. The BTVs were defined as the isodensity volumes when adjusting different percentage of the maximal threshold levels, excluding any noise or artifact from surrounding normal tissues, including brain, extracting teeth pocket, or pharyngeal constrictors. The percentage threshold was adjusted from 10% to 50% with interval of 5%, and the BTVs were determined for each threshold. The interval of threshold change could be further reduced to 1% for achieving the best fitness of the defined C-pGTV from both the tumor volume and the morphology. To simplify the volume analysis, only signals overlying the pGTV were chosen. The volumetric data of the different BTVs were automatically measured by the RTP, and this volume excluded any nGTVs. By this way, a suitable threshold level (sTL) could be defined when the morphology and the calculated BTV value using a certain threshold level was observed to be the best fitness of the volumetric data from the C-pGTV (Figure 1, 2, 3). In addition, a suitable SUV (sSUV) values were calculated as the sTL multiplied by individual SUVmax values.

## Results

### Volumetric and SUVmax data

**Figure 4:**
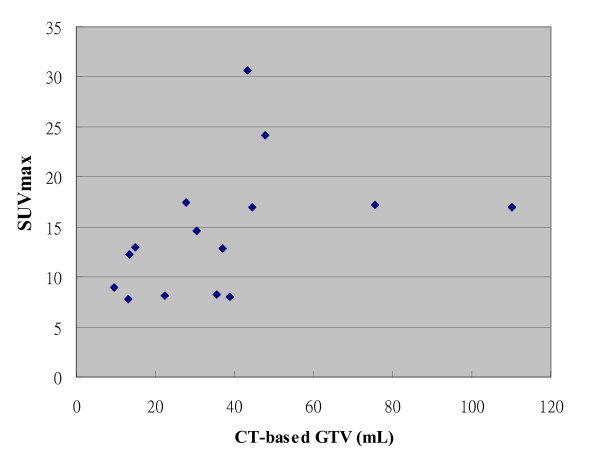
**The association between the SUVmax and the CT-based pGTV**.

Volumetric and SUVmax data for the 15 primary tumors are listed in Table 1. The volumetric data and related SUV information for the nGTVs were excluded for simplification of the study. The mean C-pGTV was 36.9 ± 26.4 mL, and the range was 9.6 to 110.2 mL, whereas the mean maximum tumor diameter in any direction on CT was 4.33 ± 1.01 cm, and the range was 3.2 to 6.3 cm. The mean SUVmax was 13.98 ± 6.4 with the range of 7.8 to 30.6. As listed in Table 1, the BTV values at different threshold level showed an inverse correlation with increasing threshold level. In addition, there was no obvious association between the SUVmax and the C-pGTV values in our patient cohort (Figure 4). Also, there was no correlation between the maximum tumor diameter and the SUVmax.

### Correlation of sTL with C-pGTV and SUVmax

**Figure 5:**
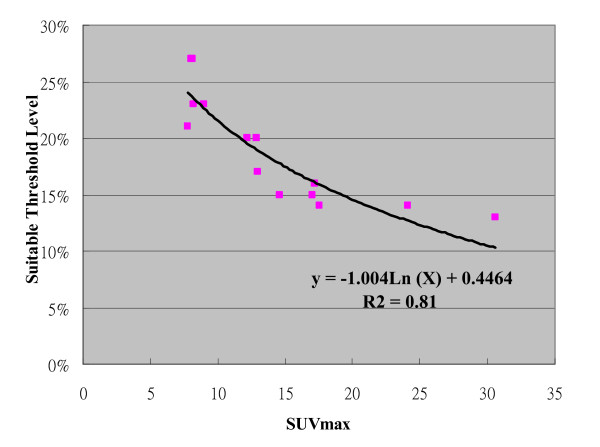
**The correlation curve between the suitable threshold level and the SUVmax**.

**Figure 6:**
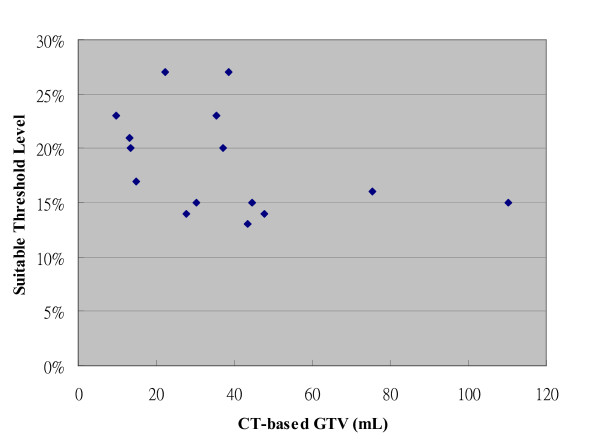
**The association between the suitable threshold level and the CT-based GTV**.

**Figure 7:**
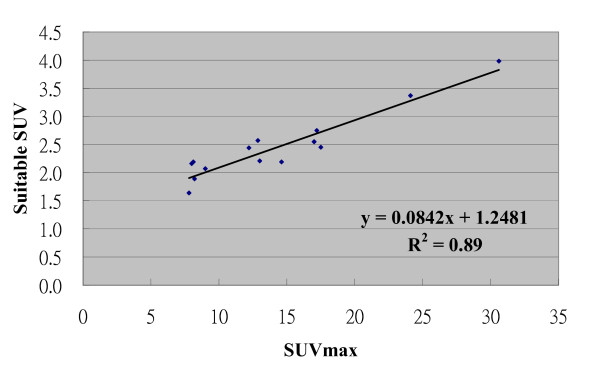
**The correlation curve between the suitable SUV and the SUVmax**.

Table 1 also showed there was no demonstrated single sTL or sSUV method for achieving optimized volumetric match with C-pGTV. For all patients, the sTL for the best match was 13% to 27% (mean, 19%; standard deviation, 4.7%). The sSUV was 1.64 to 3.98 (mean, 2.46; standard deviation, 0.58). The sSUV method of applying an isodensity volume of SUV > 2.5 failed to provide successful delineation in 60% of cases. The relation between the sTL and the SUVmax is illustrated in Figure 5. The plot illustrated an inverse hyperbolic curve with increasing SUVmax [sTL = -0.1004 Ln (SUVmax) + 0.4464; R^2 ^= 0.81]. Conversely, the sTLs were not associated with the C-pGTVs using different correlation models as depicted in Figure 6. Furthermore, the sSUVs showed a direct proportion to the SUVmax (Figure 7, sSUV = 0.0842 SUVmax + 1.248; R^2 ^= 0.89).

When excluding 4 tumors with SUVmax < 10 or eliminating 4 cases with C-pGTV < 20 mL, both the sTLs and the sSUVs were found to have a similar pattern of correlation with the SUVmax. There was no apparent association between the sTLs and the tumor volume through stratification of different SUVmax or C-pGTV levels in our studied cohort.

### Mismatch analysis

Two direction mismatch analysis was carried out as the method described by El-Bassiouni et al. [25]. When the BTVs were determined by using their sTL, the mean value for the mismatch BTVs/C-pGTV was 15.3 ± 10.3% (range, 2.4 ~ 37.5%). In contrast, **t**he mean value for the mismatch C-pGTV/BTV was 16.2 ± 14.3% (range, 1.9 ~ 48.7%). There was no significant difference between two mismatch comparison using paired *t *test (*p *= 0.72).

## Discussion

Rothschild et al. reported a matched-pair comparison study that PET/CT staging followed by IMRT improved treatment outcome of locally advanced pharyngeal carcinoma [28]. While incorporating this biologic image, there is also a great need for delineating tumor tissue more precisely, particularly in IMRT era. Various methods for incorporating PET into the RT plan have been reported; including visual comparisons, image overlays, fusion of PET and CT images, and PET/CT simulation. Since there is less co-registration error between PET and CT using the same DICOM coordinates, PET/CT simulation is a promising modality to improve contouring accuracy for reducing the risk of geographic misses in RT planning [29,30]. However, care must be taken in implementing this new technology as many physicians concern the standard of threshold setting in ^18^F-FDG PET. This study provides an applicable way of volumetric match when selecting a suitable threshold level for CT-based GTVs which had been previously delineated by radiation oncologists. Because these tumors would be treated by RT rather than surgical resection, our methods did not reflect a technique of determining real tumor margin or volume. Although our patient number was small, the result demonstrated a suitable threshold levels can be derived from individual SUVmax values, which might correspond to an intrinsic biological nature of a tumor. Different from those investigators that suggested using a fixed threshold for contouring in HNC [10,11,24], our results showed no distinctive value for sSUV or sTL. In addition, no obvious correlation between SUVmax and C-pGTV was found and this might imply that a large tumor is not always associated with an aggressive metabolic activity within a tumor.

There are many known factors responsible for SUV measurements and therefore tumor contours: the metabolic activity, tumor heterogeneity, and tumor motion [21]. Despite the effect of tumor motion can be neglected in RT set-up for HNC patients, Poisson distribution of pixel intensity does make the use of SUVmax a less reliable starting point for tumor delineation [31]. Nonetheless, SUVmax is important biologic parameter and can be easily obtained from routine ^18^F-FDG PET image. On the other hand, the only investigation published to date on the use of a source-to-background algorithm in patients focused on larynx tumors [32]. In the chest, mean ^18^F-FDG uptake in normal tissues may vary between a SUV of < 1 (lung) up to a SUV of > 3 (liver) [20]. In the head and neck region, higher SUV area can be observed in adjacent brain, Waldeyer's ring, extracted teeth pocket, pharyngeal constrictors, and vocal cord region. Thus, it is required to carefully subtract any tumor-unrelated artifacts from these areas when delineating the BTV.

Black et al. reported the results of a phantom experiment designed to evaluate the role of mean target SUVs in conditions of various target-to background ^18^F-FDG activities [31]. They showed that the threshold SUV was linearly correlated with the mean target SUV [threshold SUV = 0.307 × (mean target SUV + 0.588)]. Theoretically, it might be more ideal to use mean target SUV instead of SUVmax for threshold analysis since mean target SUV could characterize an average uptake value of certain tumors. However, the volume of the GTV must be identified first to obtain a mean target SUV. This method may be feasible for a known-sized phantom but not for real tumors whose contours are susceptible to the inter-observer variances.

El-Bassiouni et al. reported a pilot study to define the best threshold of ^18^F-FDG uptake for tumor volume delineation of HNC [25]. By using the background-subtracted tumor maximum (THR) uptake for PET signal segmentation, they found an inverse correlation between the threshold of THR and the tumor maximum uptake (S), but no correlation between the threshold of THR and the ratio of tumor maximum uptake to the background uptake (S/G). They also suggested a threshold of THR of 20% in tumors with S > 30% kBq/ml and 40% with S < 30% kBq/ml. The correlation between the threshold of THR and the S was a novel finding; however, for those PET centers using SUV for counting FDG-avid tumor uptake, direct measurement of the maximum uptake values might be not always practicable.

Schinagl et al. compared five methods for determining the BTV using coregistered CT and FDG-PET in HNC patients [26], including visual GTV, 40% and 50% of SUVmax, an absolute SUV of 2.5, and an adaptive threshold based on the signal-to-background ratio. The clinical implications from their studies were two folds. First, an isodensity volume of SUV > 2.5 failed to provide delineation in 45% of cases, which was similar with our finding. Second, PET frequently detected substantial tumor extension outside the CT-based GTV (15-34% of PET volume). The rate was also comparable with our result that the mean value for the mismatch BTV/C-pGTV was 15.3 ± 10.3%. Theoretically, the mismatch is somewhat attributed to the limitation of voxel density or a partial volume effect. In practice, it is hard to exactly define the real tumor volume outside CT-based GTV from PET image without surgical intervention. However, contouring accuracy can be improved further if radiation oncologists evaluate accordingly the change of BTV by adjusting different threshold levels during contouring.

Our study failed to show an inverse correlation between sTLs and C-pGTVs as the threshold study reported by Biehl et al. in lung cancer [21]. Using the similar method, they found optimal threshold was inversely correlated with CT-based GTV (R^2 ^= 0.79). The optimal threshold level in their study was 24 ± 13%, compared to that of 19 ± 4.7% in our study. This discrepancy might be attributed to two explanations. First, the SUVmax in their data was in direct proportion to the increase of maximum tumor diameter, which was not observed in our result. Probably, reduction of optimal threshold could be anticipated following the increase of tumor volume or Smax. Second, the measured tumor volumes in their study were far larger than those of our data (mean tumor volume: 198 ± 277 mL vs. 36.9 ± 26.4 mL). The difference might not only represent the dissimilar clinical situation when irradiating two types of cancers, but perhaps contribute to the diverse experimental findings. Of course, more investigations are required to elucidate the biological difference of the two cancers in ^18^F-FDG PET/CT image.

In another study described by Nestle et al., they analyzed various modalities for determining the BTV for lung cancer, including visual GTV, 40% of SUVmax, an absolute SUV of 2.5, and tumor-to-background ratio [20]. They found substantial differences of up to 41% among these 4 different methods. They concluded that the 40% threshold method was not suitable for target volume delineation. Based on the results of our study and other reports [20,21,24,25], a fixed threshold model is questionable in tumor volume delineation because it relies mainly on the uniformity of SUVs within the tumor. Theoretically, a unique threshold setting may fail to adequately model the lack of uniformity of ^18^F-FDG uptake because of factors such as hypoxia and necrosis, which are more likely to occur in large tumors or tumor with a higher SUVmax. For other BTVs with higher threshold than sTL, these metabolically active areas might be useful in assigning dose intensification during IMRT. Of course, the medical significance of including these additional data in the original treatment plan on final patient outcome is yet to be determined.

There are several limitations in our study. First, there was no reason that the metabolic activity should be definitely related to the real tumor volume. Undoubtedly, a surgical study must be done to answer the question. Also, the C-pGTV, used as reference image in the present study, could identify areas not strictly related to tumor tissue. Third, it is imperative to clarify whether the results could be reproducible when the same patients were scanned at different time even if their serum glucose levels were normal before images. Finally, the results have to be tested on another cohort of HNC patients to see how well the correlation equations were working. Certainly, a validation study is ongoing to reconfirm our preliminary finding.

In conclusion, a suitable threshold or SUV level can be established by an adaptive approach by correlating with SUVmax rather than using a fixed value. It will be a subject of our future work to correlate the threshold with more tumor-related factors, such as hypoxia, proliferation and histological difference. In PET-based RT planning for HNC, careful selection of a suitable threshold is imperative because this value is required to adequately encompass tumor without compromising adjacent normal tissues.

## Competing interests

The authors declare that they have no competing interests.

## Authors' contributions

CHK and SWC are responsible for the study design, coordination and drafted the manuscript. TCH, YCY and KYY collected the PET/CT data and performed analysis. SWC, SNY, YCW and JAL were responsible for the evaluation of the patients and the collection of clinical data. CRC provided some intellectual recommendation and reviewed the manuscript. CHK and SWC wrote the final version of the manuscript. All authors read and approved the final manuscript.
